# Percutaneous Cerclage Wiring Combined with Cephalomedullary Nailing for Irreducible Subtrochanteric Fractures

**DOI:** 10.1111/os.13144

**Published:** 2021-08-25

**Authors:** Shi‐Jie Kang, Fei‐Long Bao, Dong‐Sheng Huang, Tao Jiang, Yi‐Ming Hu, Jian‐Min Li, Tao Liu

**Affiliations:** ^1^ Department of Orthopedic Surgery Qilu Hospital of Shandong University Qingdao China; ^2^ Department of Orthopedics Qilu Hospital of Shandong University Jinan China

**Keywords:** Cephalomedullary nailing, Clinical results, Internal fixation, Percutaneous cerclage wiring, Subtrochanteric fractures

## Abstract

**Objective:**

To explore the surgical method, operation essentials and the clinical effect of the treatment of irreducible subtrochanteric femoral fractures by percutaneous cerclage wiring and Cephalomedullary nail.

**Method:**

From February 2016 to October 2019, 17 cases of irreducible subtrochanteric femoral fractures (SFFs) treated via a minimally invasive wire system and intramedullary nail fixation were reviewed retrospectively. Ten male and seven female patients were involved. The average age was 59.88 ± 16.13 years, ranging from 41 to 94 years. Among the patients, seven were injured in traffic accidents, five fell from a standing height, and five injured themselves from falling. The cases were classified based on the Seinsheimer classification. Specifically, five cases were type IIIA, five cases were type IIIB, one case was type IV, and six cases were type V. According to the AO/OTA classification, 10 cases were 32B3, and seven cases were 32C3. During surgery, the patients were placed on a traction bed andattempted closed reduction. For those patients whose closed reduction failed confirmed by fluoroscopy, we performed a small anterolateral incision through which a self‐made minimally invasive percutaneous wire introducer (passer; patent Z: 2016 2 1002800.8) was employed for temporary fixation with a wire. A double‐stranded steel wire was introduced into a self‐made wire traction and lifting device (patent ZL 2020 2 0205658.7), the wire was pulled vertically and firmly fixed. Then an long InterTan nail was used for the fixation. The following information was recorded: (i) length of the invasive incision, (ii) blood loss on the third day after surgery, (iii) operation time; and (iv) maximum displacement and angulation of the fracture ends of the x‐rayed front and side fractures before and after surgery and the maximum displacement and formation of the three‐dimensional CT‐scanned fracture ends in the coronal plane, sagittal plane, and cross section before and after surgery.

**Result:**

A total of 15 of the 17 patients were followed for 12 to 24 months. The 15 patients recovered, but one died from pulmonary infection 1 year after surgery. In the postoperative X‐ray and three‐dimensional CT observation reduction treatment, fracture displacement was less than 5 mm, each plane angle was less than 10 degrees, and postoperative fracture healing time was 3 to 14 months, with an average of 4.19 ± 4.04 months. The postoperative Harris hip function score ranged from 66 to 95 points, with an average of 80.81 ± 9.67 points. In terms of clinical outcomes, 11 cases were excellent, four cases were satisfactory, and one case was fair.

**Conclusion:**

For refractory subtrochanteric fractures, percutaneous wiring combined with Cephalomedullary nail fixation is a minimally invasive, rapid, and effective method, which can achieve satisfactory results in clinical practice and is worth promoting.

## Graphical abstract

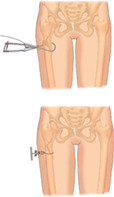

We designed and manufactured a wire minimally invasive system, including minimally invasive percutaneous wire introducer(Passer) and wire traction device. We used Passer to implant the double steel wire into the appropriate position of SFFs, and used a wire traction device to reduce the fracture distance and increase the quality of fracture reduction. The steeling traction device can provide the first compression fixation when pulled in the process of vertical fracture line and the second pressure fixation when it was tightened.

## Introduction

Subtrochanteric femoral fractures (SFFs) account for approximately 7% to 34% of hip fractures^1^, ^2^, ^3^. This region of subtrochanteric femoral is defined between the lesser trochanter and 5 cm diatal to the lesser trochanter. It is subjected to some of the highest compressive and tensile forces in the body. Because of the instability of bone fragments and the stretching and pulling forces of the muscles, the reduction and stable fixation of SFFs is still challenging^4^, ^5^, ^6^, ^7^, ^8^ for surgeons.

Over the past 20 years, major changes have occurred in the treatment of SFFs, various internal fixation materials have emerged and numerous of intra‐ and extramedullary implants have been described for internal fixation of the SFFs. However, the mainstream method remains reaming and anterograde intramedullary nail fixation^9^, ^10^. Because of a short proximal fragment with a fixed flexion, abduction and external rotation deformity, the closed reduction of these fractures cannot be completed, especially if there are long oblique or spiral bone fragments on the medial side. Cephalomedullary nailing commonly results in varus malalignment and iatrogenic displacement of fracture involving the nail entry^11^. Malreduction is regarded the most important reason of SSFs nonunion. The acceptable reduction is defined as maximum angulation in any plane of <10 and <4 mm of cortical displacement by Baumgaertner^12^,but it is difficult for close reduction of irreducible subtrochanteric fractures. Cerclage wiring for treatment of subtrochanteric femoral fractures with long oblique or spiral fracture lines has been regarded as a good surgical technique to minimize fracture gap and improve fracture stability^11^, ^13^, ^14^, ^15^, ^16^, ^17^. The long oblique or spiral fracture fragment on the medial side is usually pulled inward and proximal by strong muscle. Cephalomedullary intramedullary nails can fix the femoral head and the distal femur, but they cannot effectively fix the medial fragments of SFFs. Open reduction and Cerclage wiring can reduce the facture displacement, angulation and increase the quality of reduction^14^. Once the medial fragment reconstruction is complete, the intramedullary nailing load is reduced and the chance of implant failure is reduced. However, how to minimally use invasive wire placement and effective reduction and fixation is still insufficient in clinical studies. Kim^11^ provided the technique of percutaneous wire introduction, but there is no literature suggesting how to apply minimally reduction and fixation with invasive wire.

We designed and manufactured a wire minimally invasive system, including minimally invasive percutaneous wire introducer (Passer) and wire traction and lifting device. We used Passer to import the double steel wire into the appropriate position of SFFs, and used the wire traction and lift device to reduce the fracture distance and increase the quality of fracture reduction. The steeling traction device can provide the first compression fixation when pulled in the process of vertical fracture line and the second pressure fixation when it was tightened. This study retrospectively summarizes and analyzes the surgical method and treatment results of 17 SFF cases treated in our department *via* minimally invasive wire cerclage(Passer), wire traction and tighting devices, and Cephalomedullary nail fixation. The purpose of this study is to: (i) research the influence of steel wire cerclage on the blood supply of the femur; (ii) explore the surgical indications of minimally invasive wire‐assisted fixation and Cephalomedullary nailing for the treatment of SFFs: (iii) summarize the surgery performance of minimally invasive wire introduction and a screwing traction and tigting device in minimally invasive surgery; and (iv) to summarize the clinical effect of minimally invasive wire introduction and a wire tration and tighting devices combined with Cephalomedullary nail internal fixation for the treatment of SFFs.

## Materials and Methods

### Inclusion and Exclusion Criteria

The inclusion criteria are as follows: (i) diagnosis of closed subtrochanteric fracture for adults; (ii) long oblique or spiral fractures on the medial in which closed reduction failed; (iii) percutaneous wiring combined with Cephalomedullary nail fixation; (iv) postoperative follow‐up over 12 months and (v) acute injuries (<3 weeks).

The exclusion criteria are as follows: (i) pathological fractures; (ii) open fractures; (iii) patient follow‐up examinations less than 12 months; and (iv) patients with other diseases who cannot tolerate surgery.

This study was approved by the ethics committee of Qilu Hospital, and all the patients signed an informed consent form.

### General Information

This study retrospectively examined 17 patients with SFFs treated *via* percutaneous wire introduction and intramedullary nail fixation in the Department of Traumatology and Orthopedics, Qilu Hospital, Shandong University (Qingdao), from February 2016 to October 2019. This study included 11 male and seven female patients between the ages of 41 and 94 years, with an average of 59.88 ± 16.13 years. In terms of trauma and violence, seven patients were injured in traffic accidents, five were injured from falling from a certain height, and five were injured from falling. Preoperative imaging showed a subtrochanteric fracture of the femur with a long oblique or spiral bone fragments on the medial side. Based on the Seinsheimer classification^18^, five cases were classified as type IIIA, five cases were classified as type IIIB, one case was classified as type IV, and six cases were classified as type V. Based on the AO/OTA classification, 10 cases were classified as 32B2A, and 7 cases were classified as 32B3A.

### Surgical Strategy

#### Preoperative Preparation

After admission, routine blood test, blood biochemistry, and coagulation mechanisms were checked. Fracture lines were analyzed based on the anteroposterior and lateral positions of the femur and three‐dimensional (3D) CT. Routine treatment included tibial tubercle bone traction, anti‐edema treatment, and low‐molecular‐weight heparin or rivaroxaban to prevent thrombosis. In the lower extremity ultrasound examination, in the absence of lower extremity thrombosis, a lower extremity venous pump was employed to prevent thrombosis. If lower extremity thrombosis exists, then vascular surgery should be conducted. Before surgery, the length and diameter of the intramedullary nail to be used were assessed based on the measured value of the PACS system.

Anesthesia and Position Among the 17 patients, 14 were given epidural anesthesia, and three were administered general anesthesia. With the preventive use of antibacterial drugs 30 min before surgery, 1 g of tranexamic acid was injected intravenously. While lying on a traction bed, the patient's contralateral side was abducted and rotated externally to the abduction frame, the affected limb was placed on the traction bed, and feet were firmly fixed.

#### Reduction and Fixation

An assistant fixed the patient's pelvis, and another assistant flexed the patient's knees and hips, and abduction and external rotation traction. The surgeon squeezed the reduction on the anterior and posterior medial sides according to the fracture line, and the assistant maintained the abduction and external rotation traction and gradually straightened the injured limb. Next, internal rotation reduction was conducted, joints were fixed on the traction bed, reduction was maintained, fluoroscopy was conducted to check the proximal femur anteroposterior and lateral positions, and the butterfly or long spiral fractures were displaced within less than 4 mm or the proximal femur or mild valgus in the anterior position were anatomically reduced^12^. The pelvis and affected side of the lower extremity were disinfected, and the lengthened proximal femur intramedullary nail (INTERTAN, Smith‐Nephew, Austin, TX) was inserted as a standard operation. If the displacement of the butterfly or long spiral fracture segment is more than 4 mm or in the case of angular deformity, the intramedullary nail selected before the operation is placed on the anterior side of the femur. The position of the intramedullary nail was checked *via* fluoroscopy. The entry point of the head nail was selected, the local incision was separated with a periosteal dissector, the nail was placed on the inner side of the proximal fragment of the fracture, compression and rotation reduction were conducted, the butterfly or long spiral fracture was mobilized, and the steel needle was tilted. If the fracture line displacement is less than 4 mm or anatomical reduction or mild valgus is observed in any position, reduction is maintained, and the lengthened proximal femur intramedullary nail is driven in as a standard operation. If the fracture line displacement is more than 5 mm or the temporary reduction of the fracture cannot be maintained, then the wire introduction cerclage‐assisted reduction method is adopted.

**Fig. 1:**
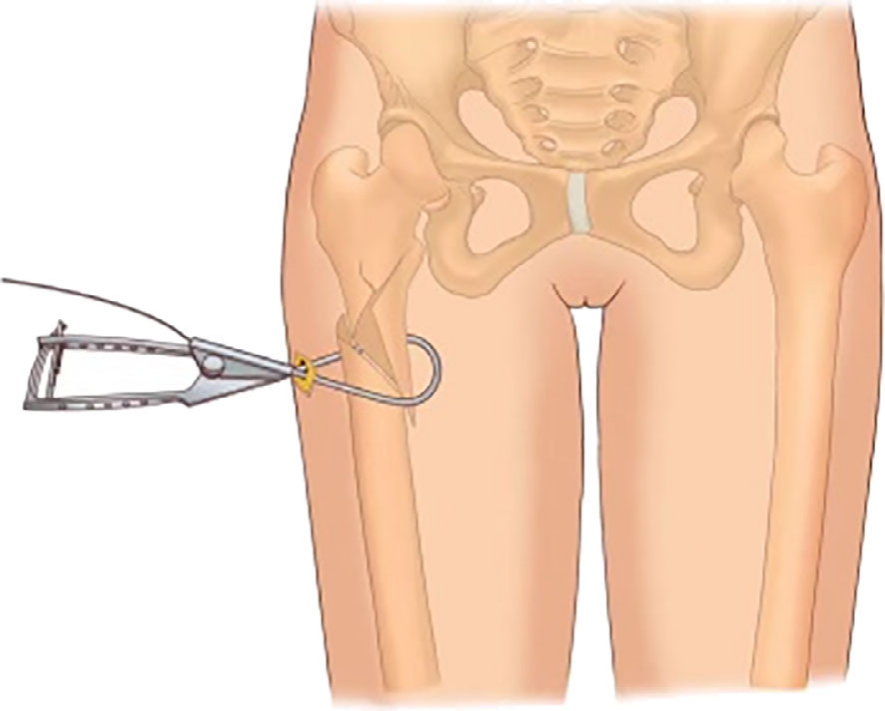
Diagram of passer.

**Fig. 2:**
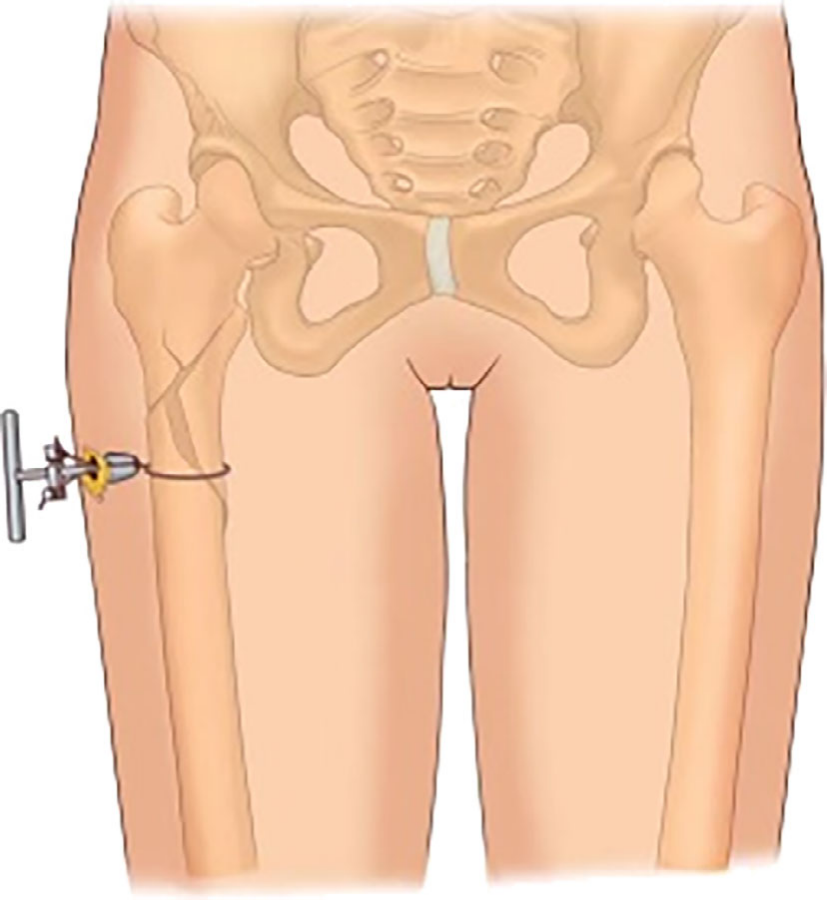
Diagram of wire traction and tighting devices.

Under fluoroscopy, according to the length and location of the fracture line, one to two local incisions approximately 3 to 5 cm long were made at the anterolateral femur. The skin and subcutaneous tissue were cut in turn, the lateral femoral muscles were separated, long curved forceps were inserted along the periosteum for separation, and the posterior femur was opened at the thick line to facilitate the placement of the wire introducer (passer, Fig. 1). The long curved forceps were used to tilt the bone block to correct the distance and flip displacement, and a separate passer was inserted in the front and back of the femur and merged. If the position of the bone block under fluoroscopy is satisfactory, then a double‐stranded steel wire is inserted through the wire guide hole, and the passer is withdrawn. The double‐stranded steel wire was led into the screwing traction pressurizer, which was then pushed along the channel to the bone surface. Next, the steel wire was pulled vertically in the fracture displacement direction under fluoroscopy, and the compression device (Fig. 2) was pushed simultaneously to promote fracture space reduction. If the fracture line disappears or is less than 4 mm, then the steel wire is fixed to the fixing knob of the pressurizer then rotated and tightened. After 3 to 4 buckles, if the fracture reduction is acceptable under fluoroscopy, then the steel wire is cut to maintain the reduction, and the extended proximal femur intramedullary nail is inserted as a standard operation.

#### Postoperative Management

(i) Antibacterial drugs were given to a patient for 24 h as a preventive measure; (ii) analgesics were given according to a patient's VAC score; (iii) anticoagulant treatment with low‐molecular‐weight heparin or rivaroxaban was initiated 12 h after surgery: (iv) drainage strips were removed 24 to 48 h after surgery; (v) the patient was instructed to perform CPM passive functional, bedside sitting, hip and knee joint flexion, and extension exercises after postoperative pain was relieved: and (vi) the X‐ray was reexamined 1 month after surgery. If calluses form, then gradual exercise with weights under the protection of a walker is recommended.

### Observation Index and Follow‐up Examinations

The length of the incision for the minimally invasive steel wire implantation and operation time (the patient was moved from the tractor to the transfer bed after surgery) were recorded, and blood loss was calculated according to the Sehat method^19^ on the third day after surgery.

The maximum displacement and angulation of the fracture ends of the X‐rayed positive and lateral fractures before and after surgery and maximum displacement and angulation of the 3D CT‐scanned fracture ends on the coronal plane, sagittal plane, and cross section before and after surgery were recorded.

The patients were reexamined 2 weeks, 4 weeks, 8 weeks, 12 weeks, 6 months, and 1 year after the fracture operation. The Harris score and fracture healing time were recorded during each follow‐up examination^20^.

### Statistical Methods

All statistical analyses were conducted using the Statistical Package for Social Sciences (SPSS) 20.0 for Windows (SPSS Inc., Chicago, IL). All *P* values are two‐sided, with a significance level of 0.05. The result of statistical data were presented as mean ± SD.

## Result

### General Result

A total of 15 of the 17 patients were followed for 6 to 24 months. All 15 patients healed, but one patient died from pulmonary infection 1 year after surgery.

### Minimally invasive incision

The length of the minimally invasive incision was 4.0–5.0 cm, with an average length of 4.58 ± 0.05 cm.

### Operative time

Operative time is defined from the placement position to the postoperative departure from the traction bed. The time is 70–150 min, with an average of 113.3 ± 22.3 min.

### Blood Loss

Blood loss was calculated based on Sehat method. we record preoperative, the third day postoperative routine blood tests and blood transfusions. The average blood loss was 523.91 ± 227.28 ml (rang from 287.60 to 998.94 ml).

The maximum displacement and angulation before and after surgery

The comparison result of the maximum displacement before and after surgery was statistically significant (*P* < 0.001, Table 1), and the maximum angulation ratio before and after the operation was statistically significant (*P* < 0.001, Table 2).

**TABLE 1:** Comparison of maximum displacement before and after surgery

	Preoperative	Postoperative	*P* value
Maximum positive displacement	15.90 ± 8.42	2.90 ± 1.54	*P* < 0.0001
Maximum lateral displacement	19.81 ± 7.16	2.97 ± 1.07	*P* < 0.0001
Maximum coronal displacement	13.07 ± 8.43	3.05 ± 1.28	*P* < 0.0001
Maximum displacement in sagittal plane	17.94 ± 8.93	3.24 ± 1.34	*P* < 0.0001

**TABLE 2:** Comparison of maximum angle of stump before and after surgery

	Preoperative	Postoperative	*P* value
Maximum positive angle	14.86 ± 8.39	2.64 ± 1.64	*P* < 0.0001
Maximum lateral angle	19.15 ± 11.23	1.42 ± 1.35	*P* < 0.0001
Maximum coronal angle	12.33 ± 8.05	1.92 ± 1.48	*P* < 0.0001
Maximum sagittal plane angle	18.99 ± 7.47	1.63 ± 1.55	*P* < 0.0001
3D maximum angle	22.25 ± 7.48	2.67 ± 2.15	*P* < 0.0001

### Healing time

Average healing time was 4.19 ± 4.04 months (ranging from 3 to 14 months).

### Harris hip function score

The Harris hip function score was used postoperatively and ranged from 66 to 95 points, with an average of 80.81 ± 9.67points. Among the cases, 11 were excellent, three were satisfactory, and o was fair. Infection, fracture nonunion and malunion, and other related complications were not observed:

Typical case 1: Male, 41 years old, with falling injury (Figs 3, 4, 5, 6, 7, 8, 9, 10, 11, 12, 13, 14, 15).

**Fig. 3:**
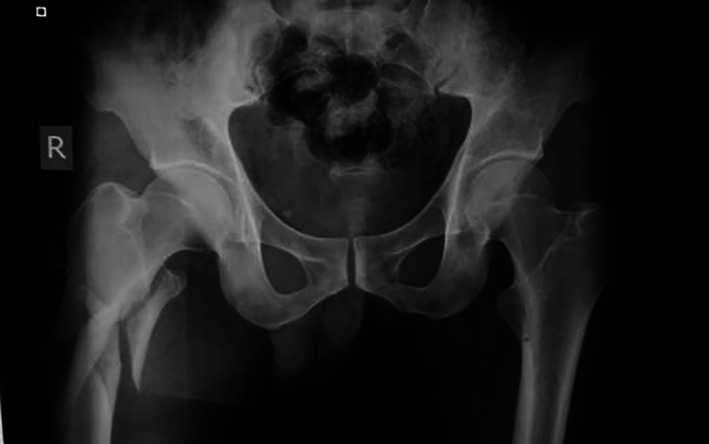
Preoperative A‐P view of pelvis.

**Fig. 4:**
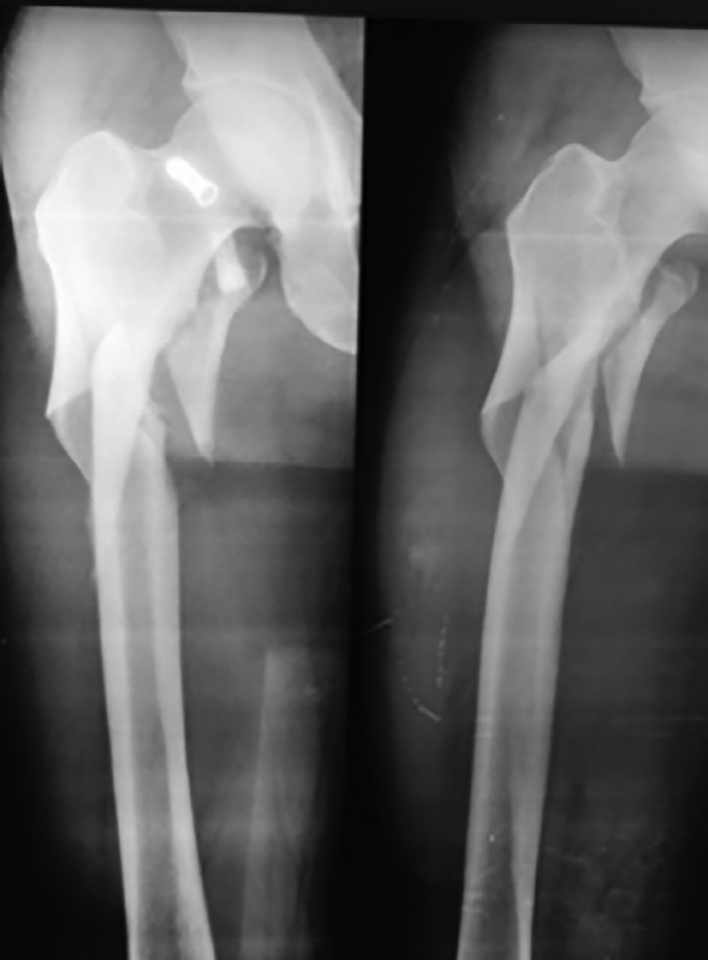
Preoperative A‐P and lateral views of proximal femur.

**Fig. 5:**
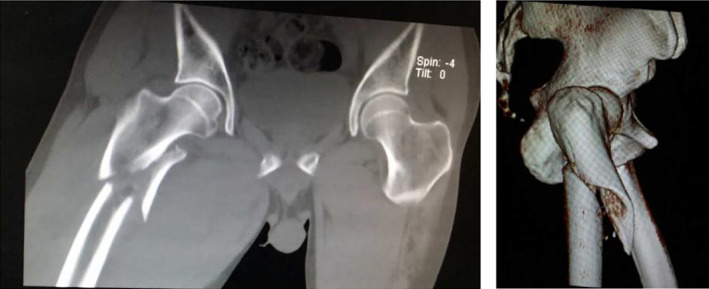
Preoperative CT and 3D scans.

**Fig. 6:**
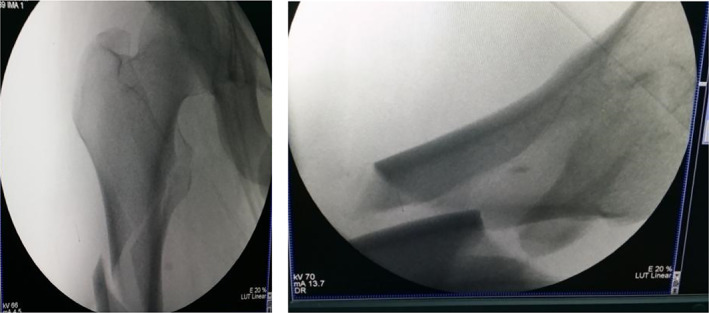
Intraoperative traction reduction; proximal femur is flexed and externally rotated, and the separation displacement exceeds 1 cm.

**Fig. 7:**
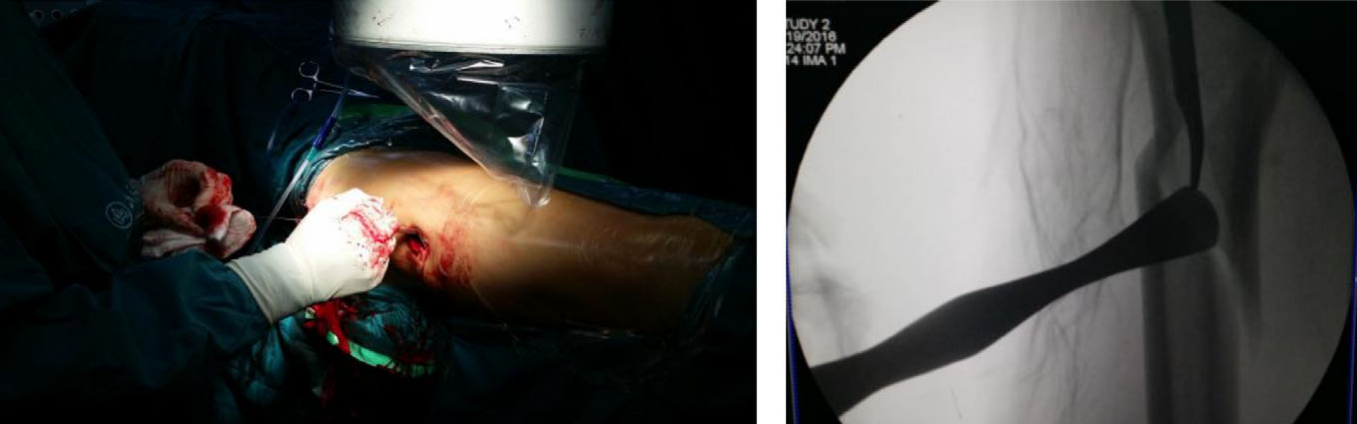
Periosteal dissector is inserted in the entry point of the head nail, the proximal end is pressed, and a finger assists in the reduction; overlapping and lateral displacement is observed in the distal end.

**Fig. 8:**
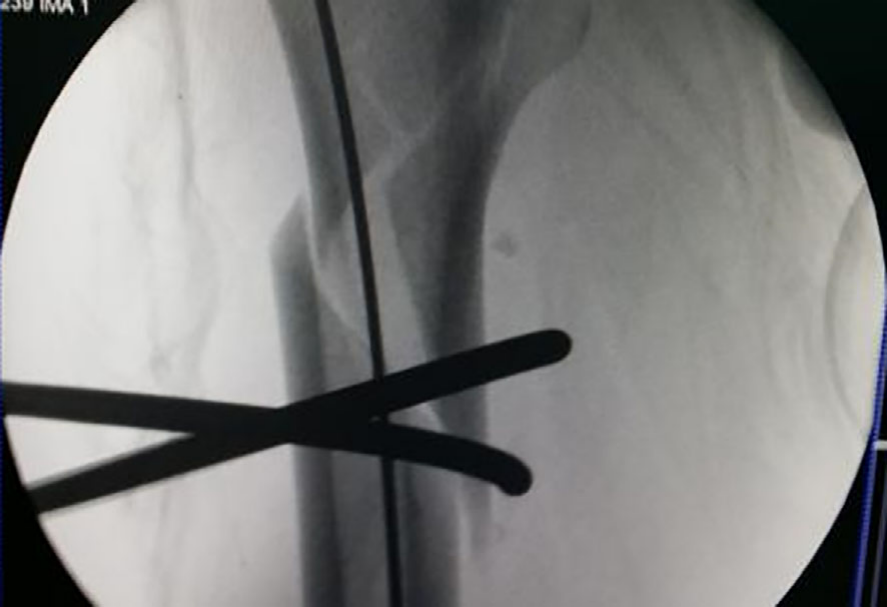
Placement of the upper and lower parts of the passer.

**Fig. 9:**
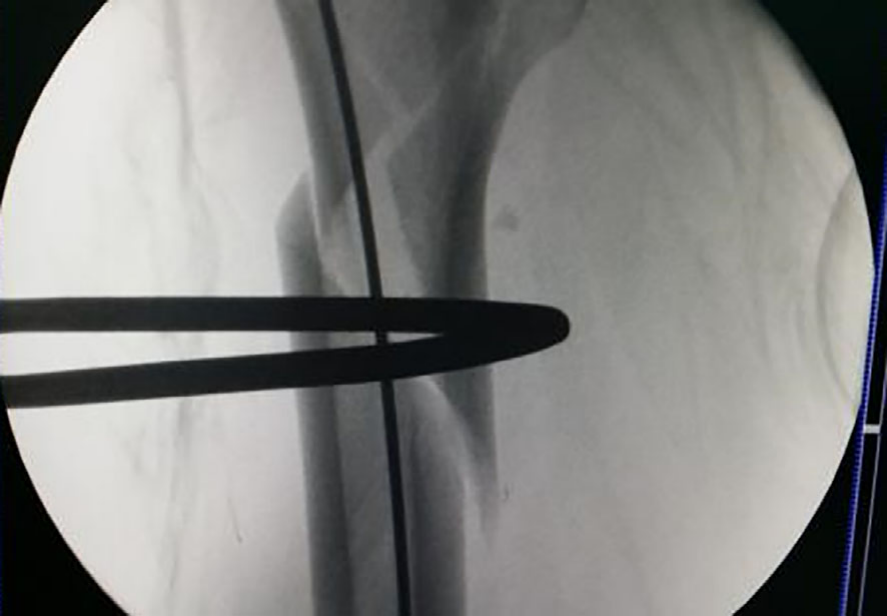
Merging of passer into a whole.

**Fig. 10:**
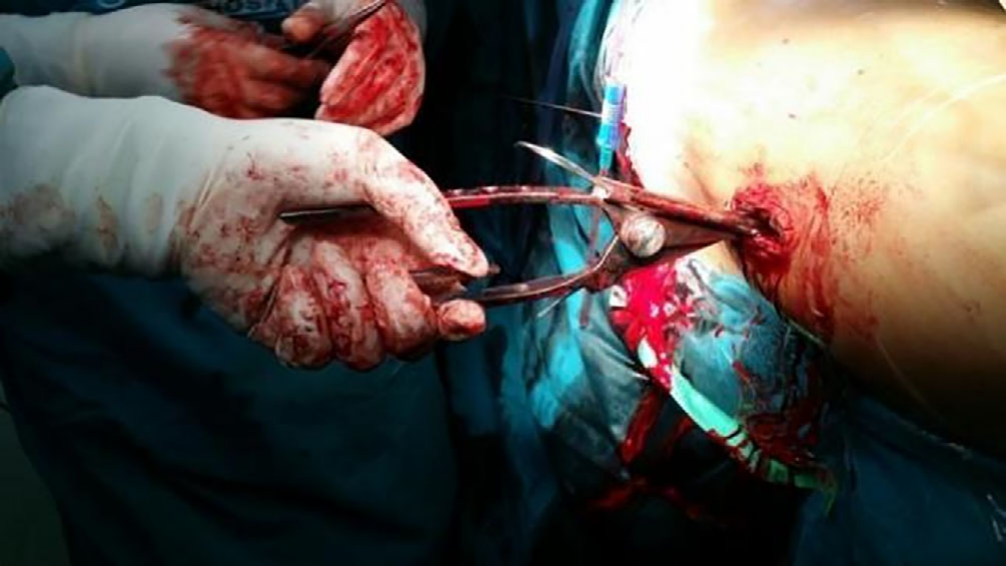
Introduce double steel wire.

**Fig. 11:**
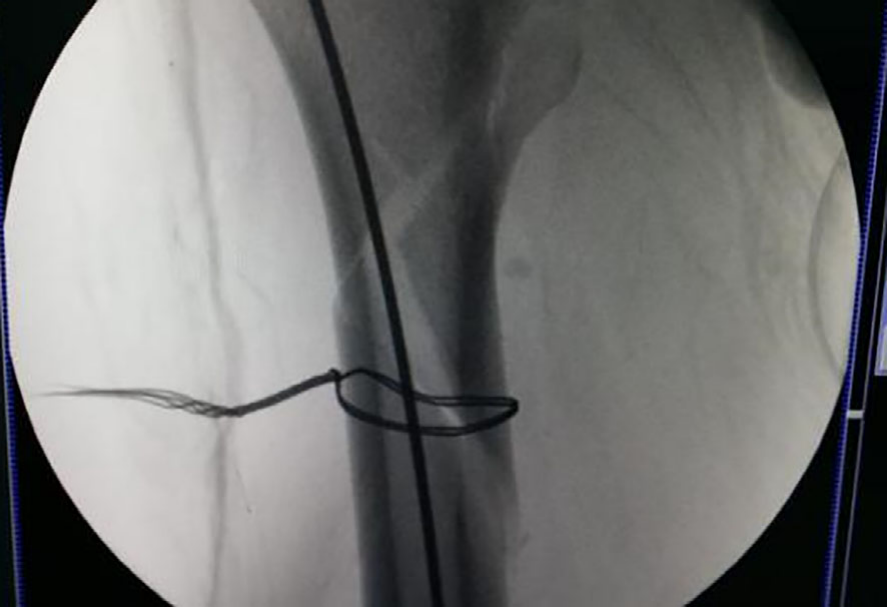
Fracture gap disappears after cerclage.

**Fig. 12:**
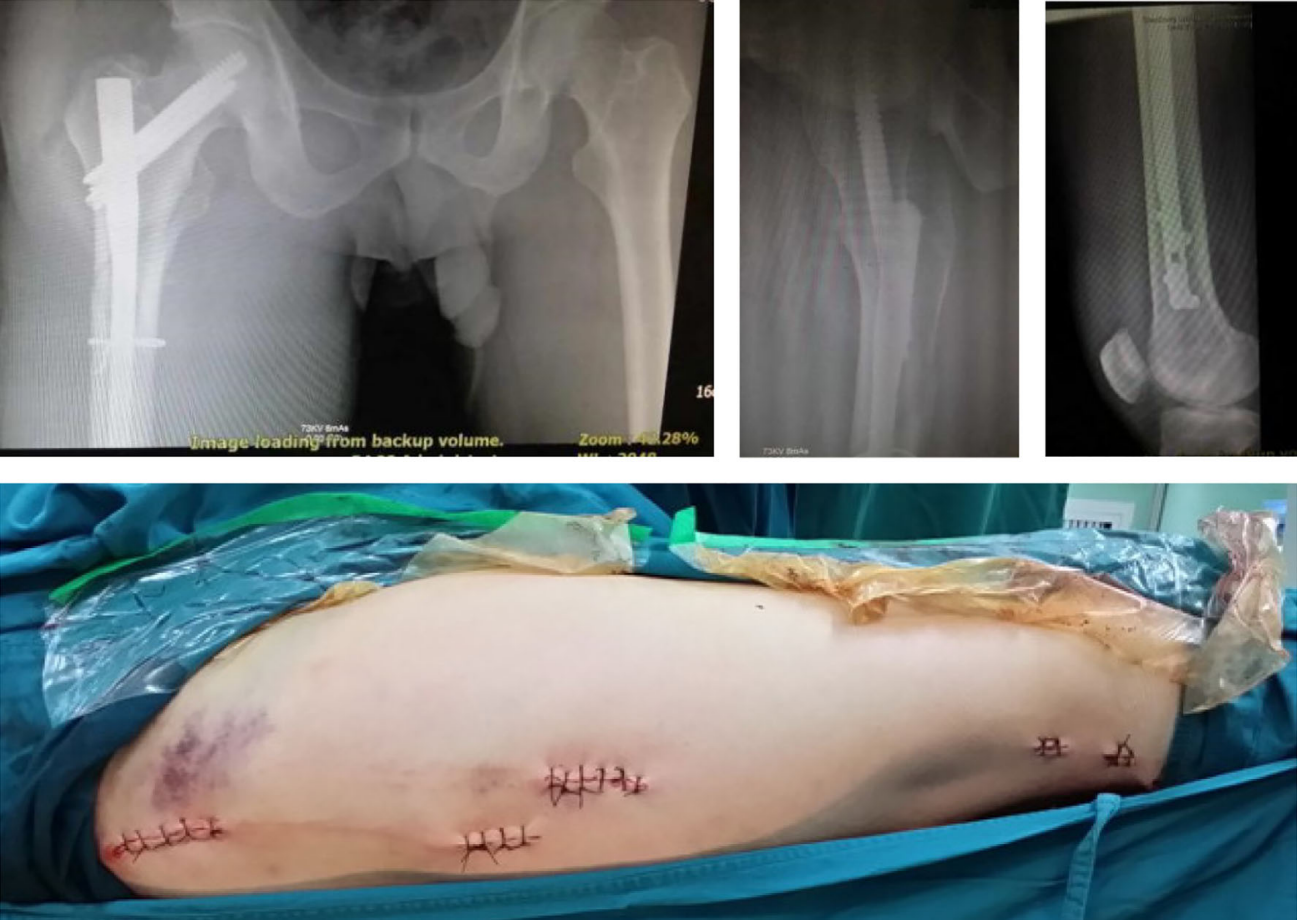
Intraoperative X‐ray showing fracture anatomical reduction and suture incision after the completion of intramedullary nail fixation.

**Fig. 13:**
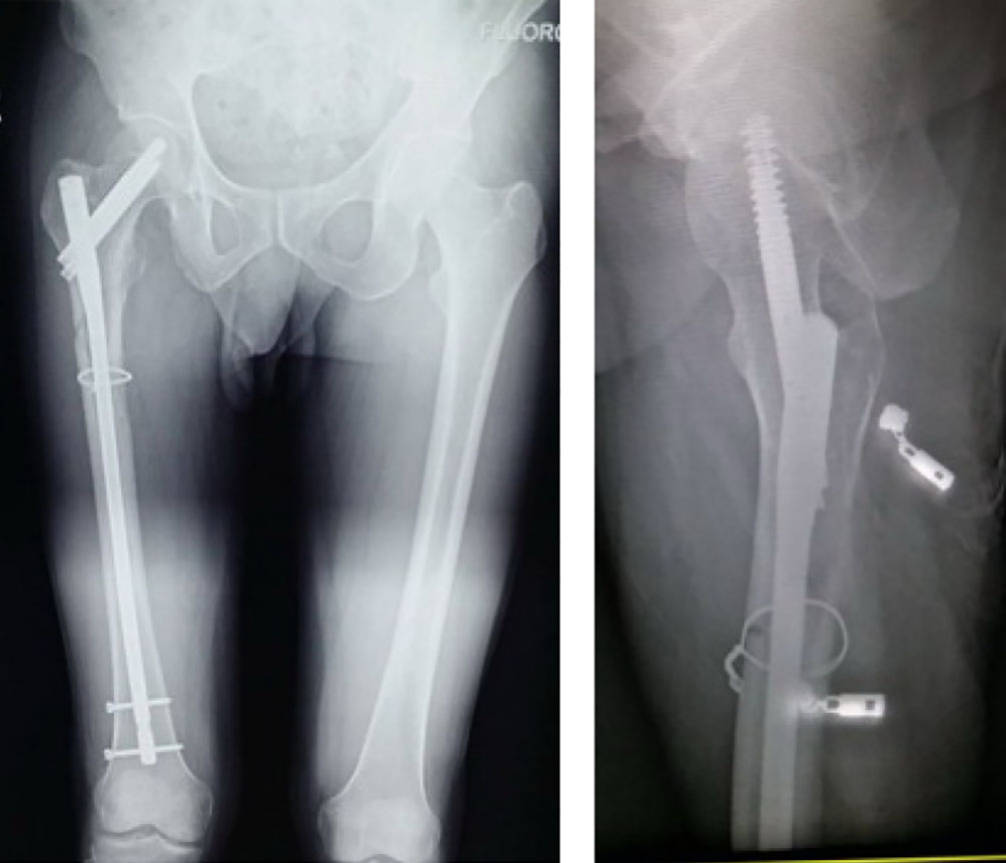
Blurred fracture line 3 months after surgery.

**Fig. 14:**
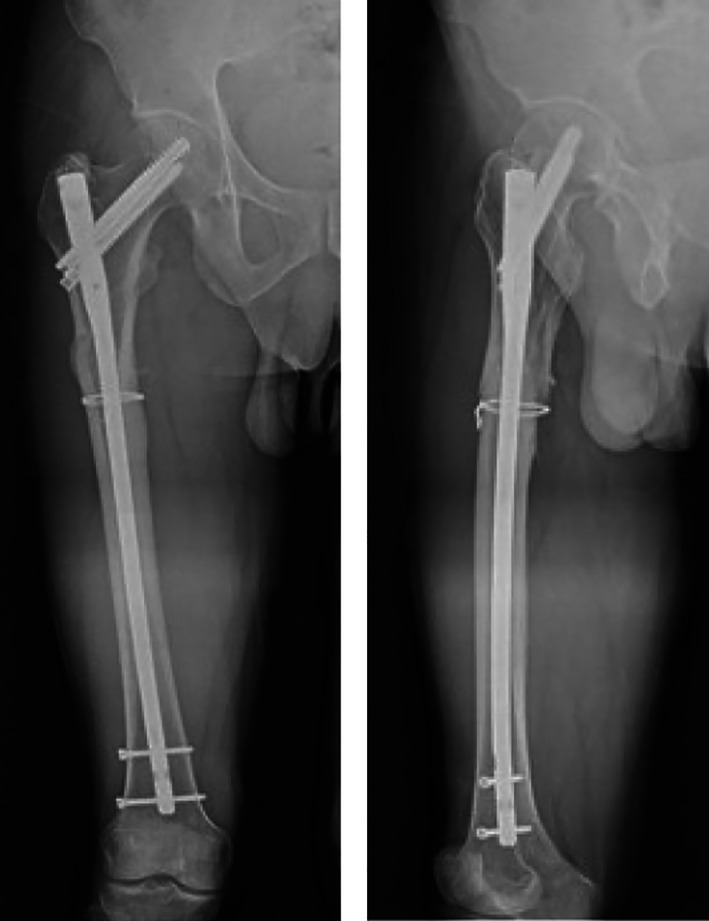
Healed fracture 1 year after surgery.

**Fig. 15:**
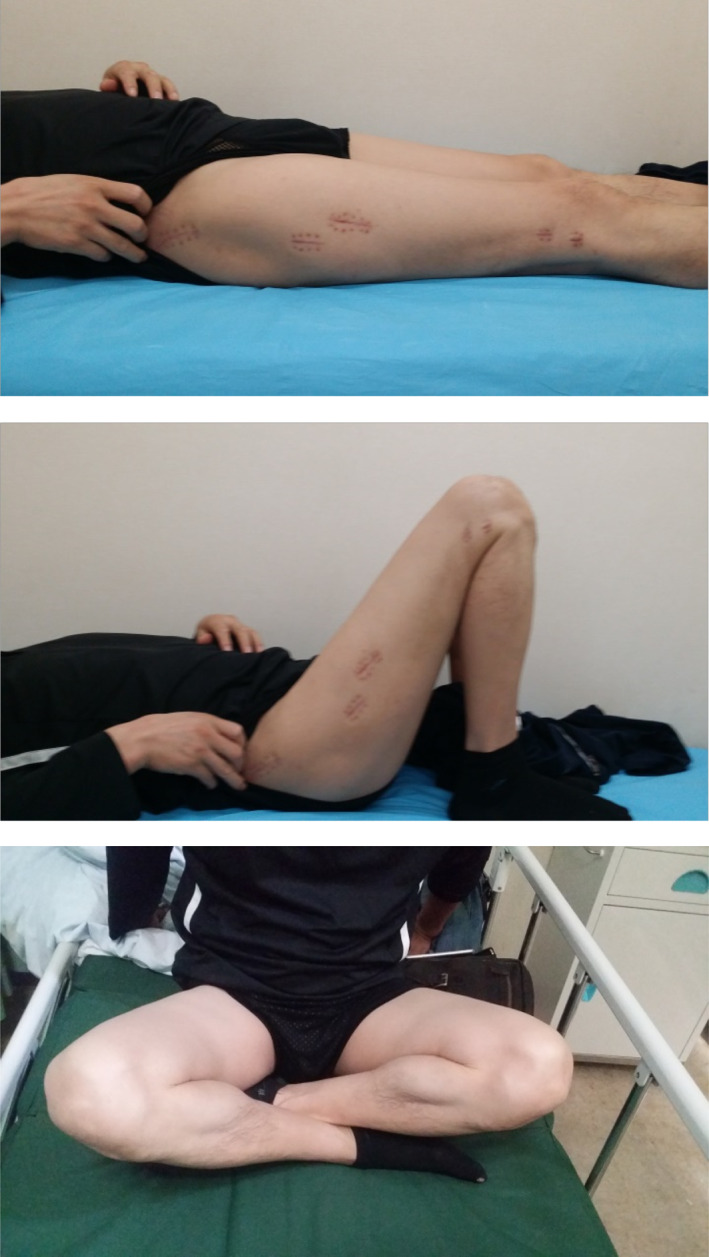
Functional results 1 year after surgery.

Typical case 2: Male, 94 years old, injury from a fall, causing SFF on right femur (AO/OTA 32B2A) (Figs 16, 17, 18, 19, 20, 21, 22, 23).

**Fig. 16:**
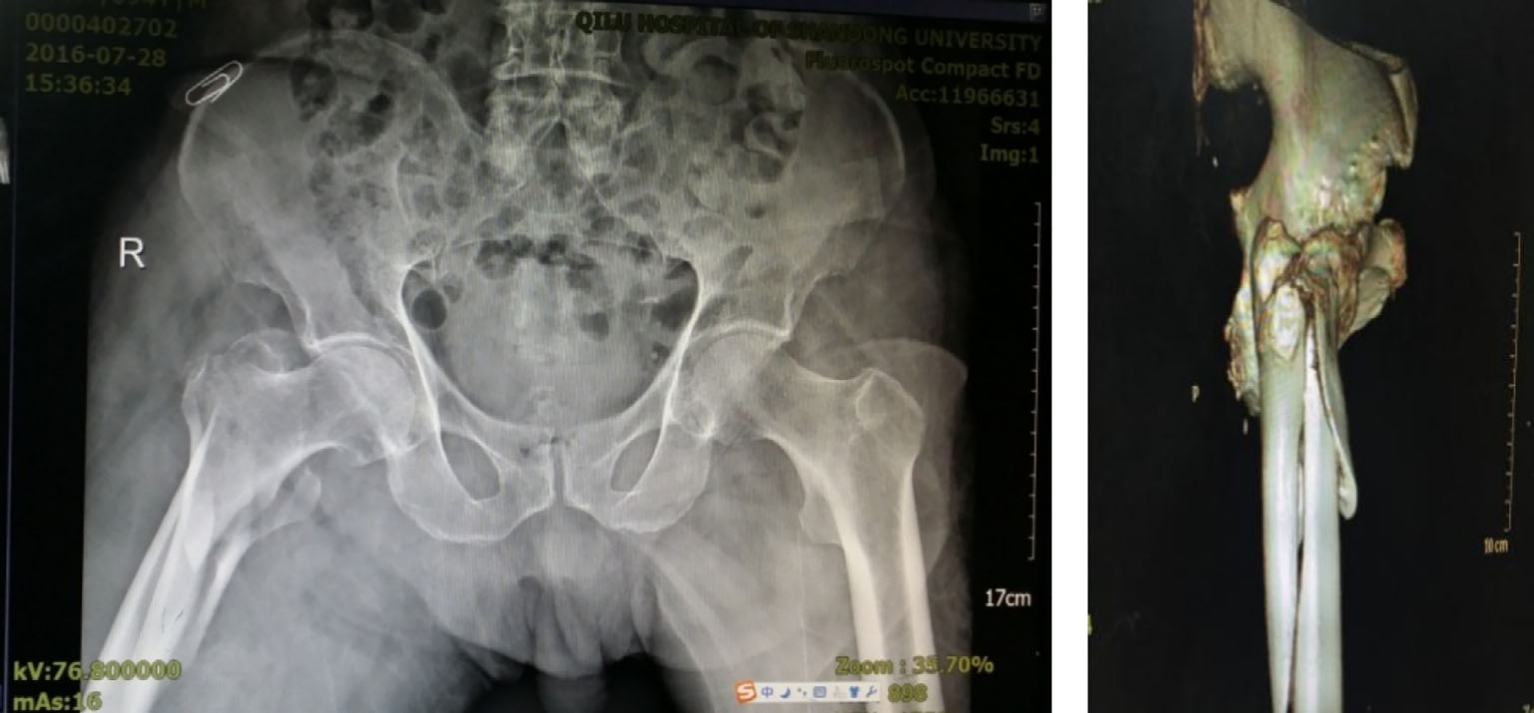
Preoperative X‐ray and CT.

**Fig. 17:**
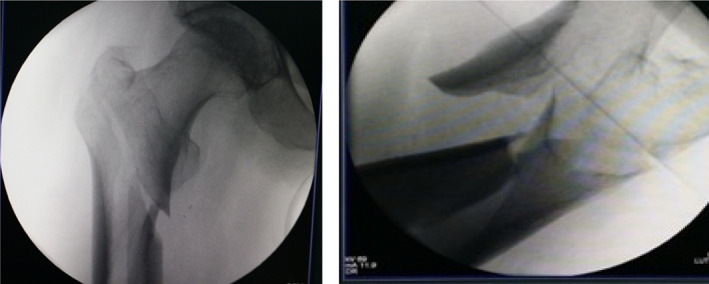
Traction reduction, but fracture displacement is obvious.

**Fig. 18:**
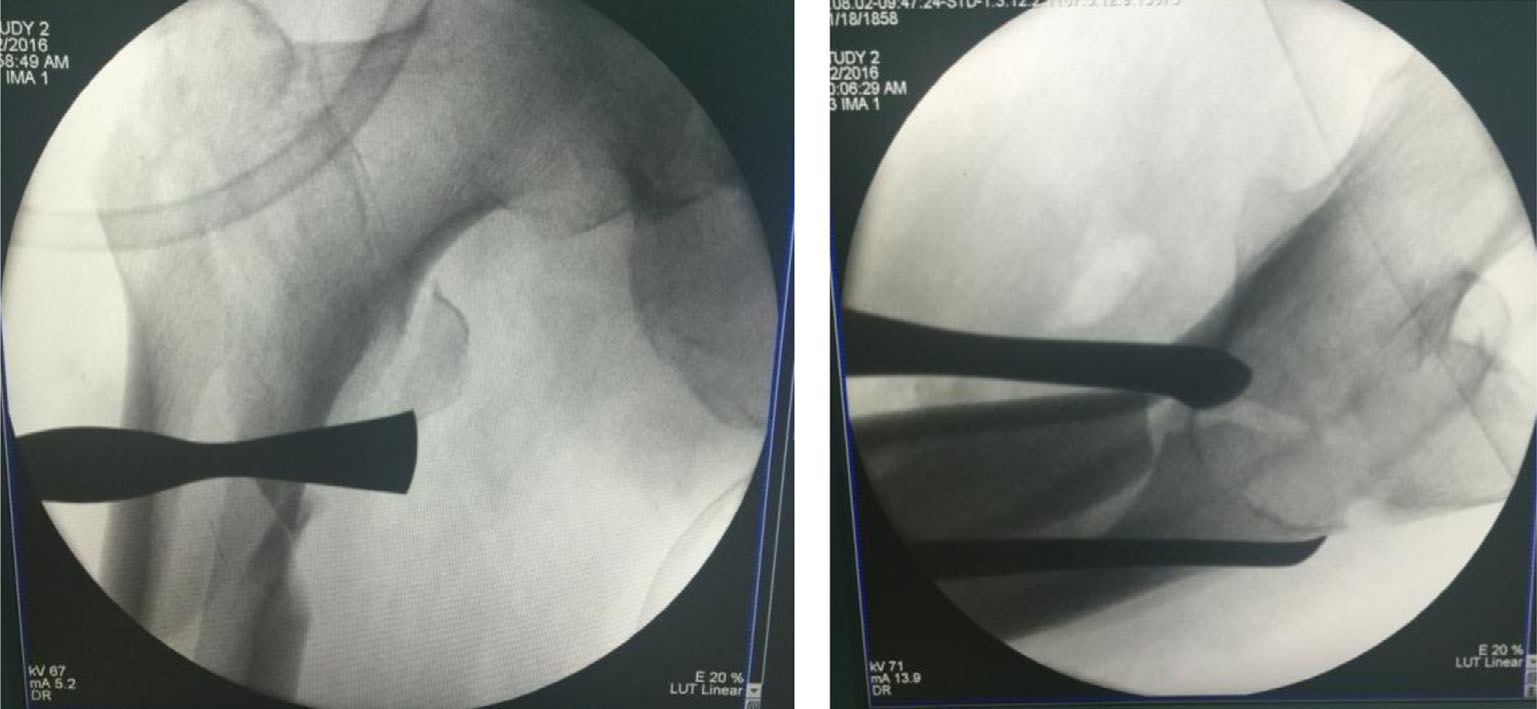
Closed reduction cannot achieve and maintain satisfactory reduction.

**Fig. 19:**
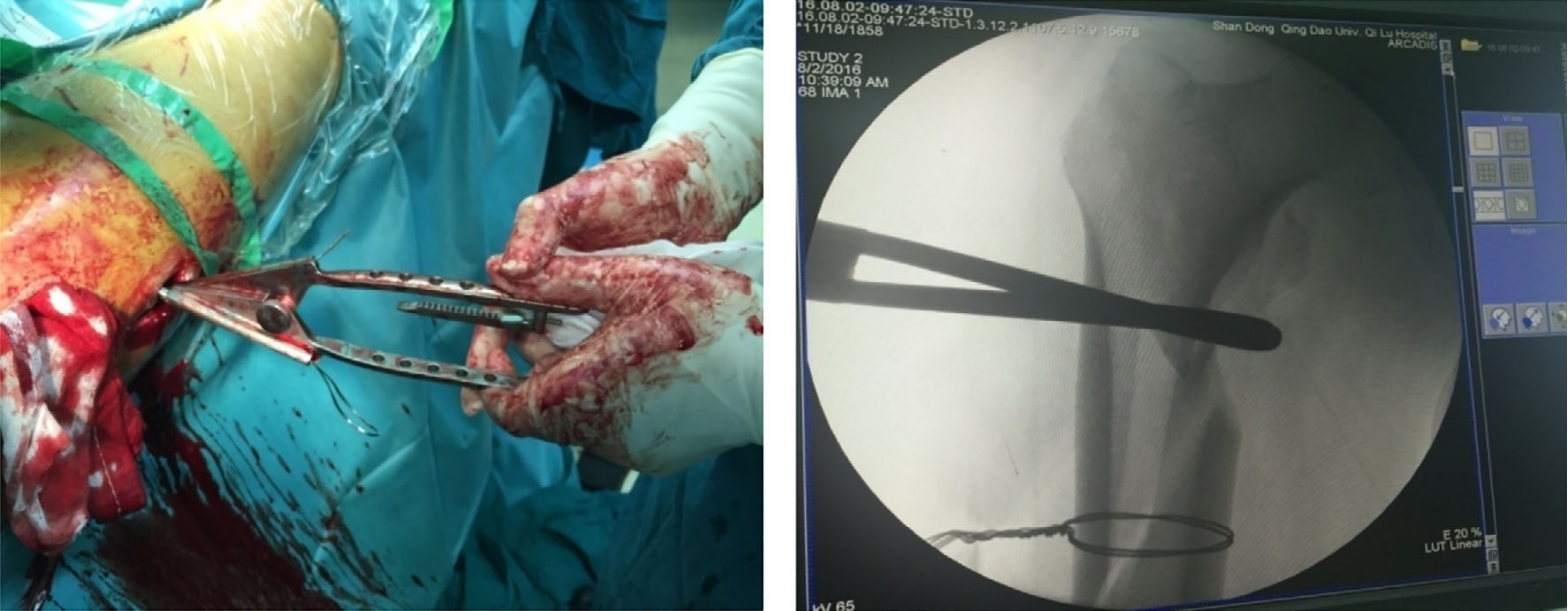
Percutaneous cerclage wiring and reduction.

**Fig. 20:**
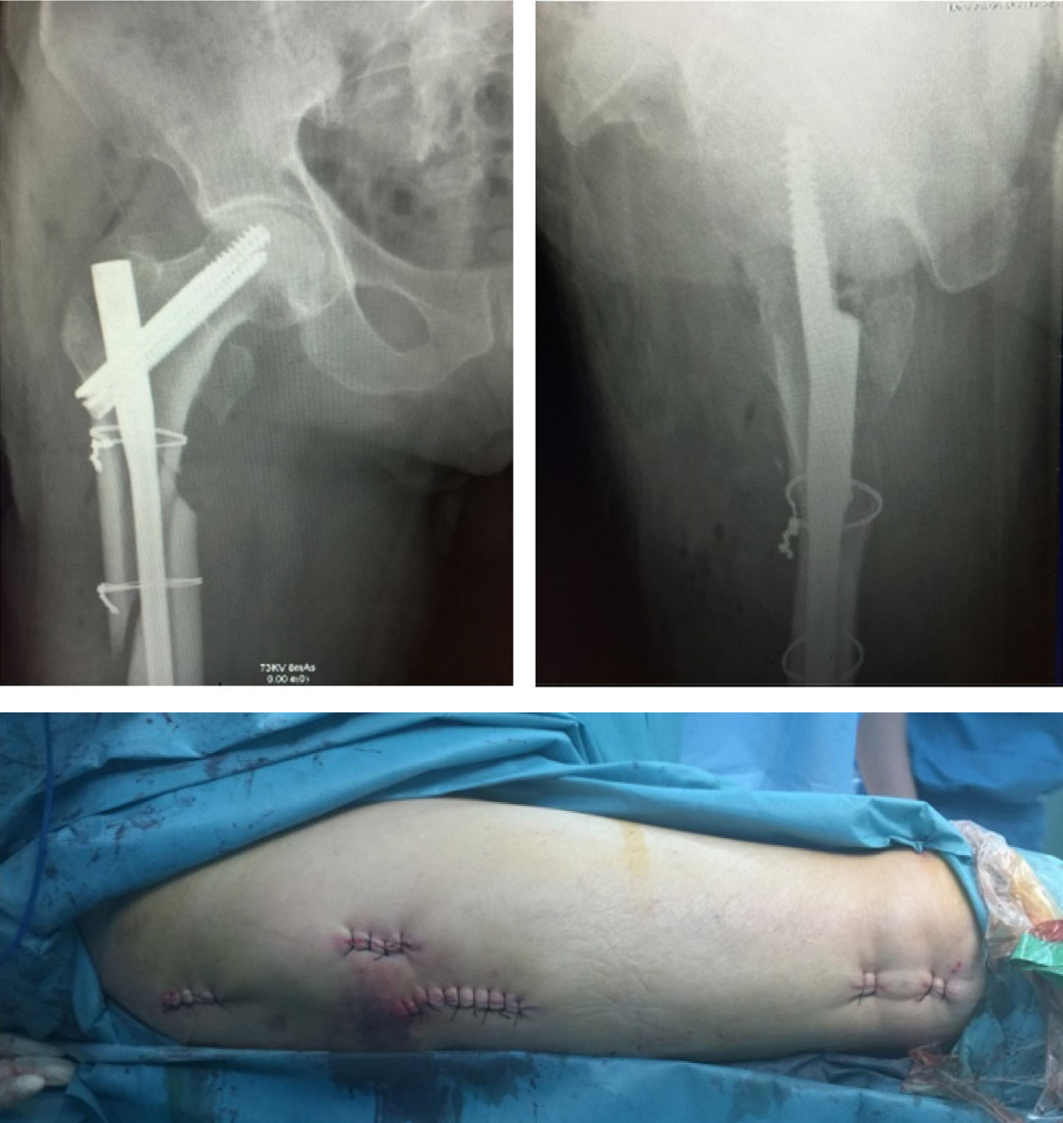
Intraoperative X‐ray and incisions.

**Fig. 21:**
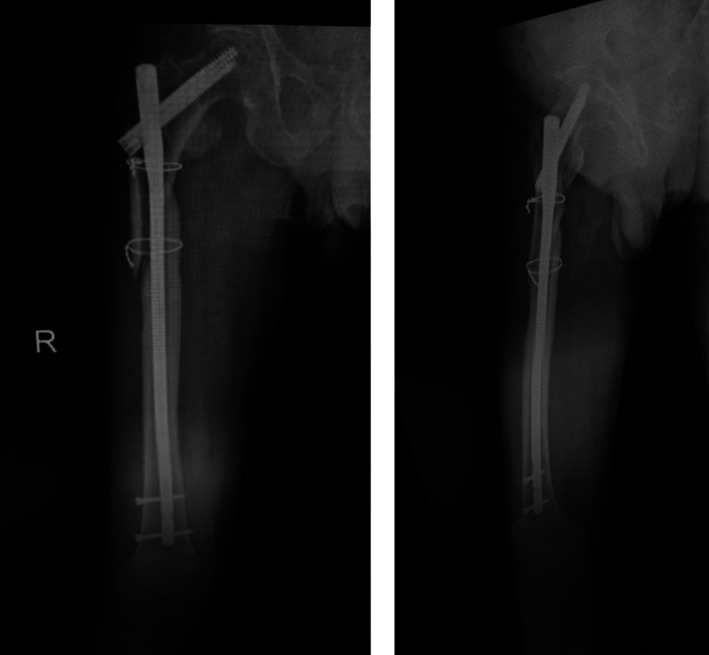
X‐ray taken 1 year after surgery.

**Fig. 22:**
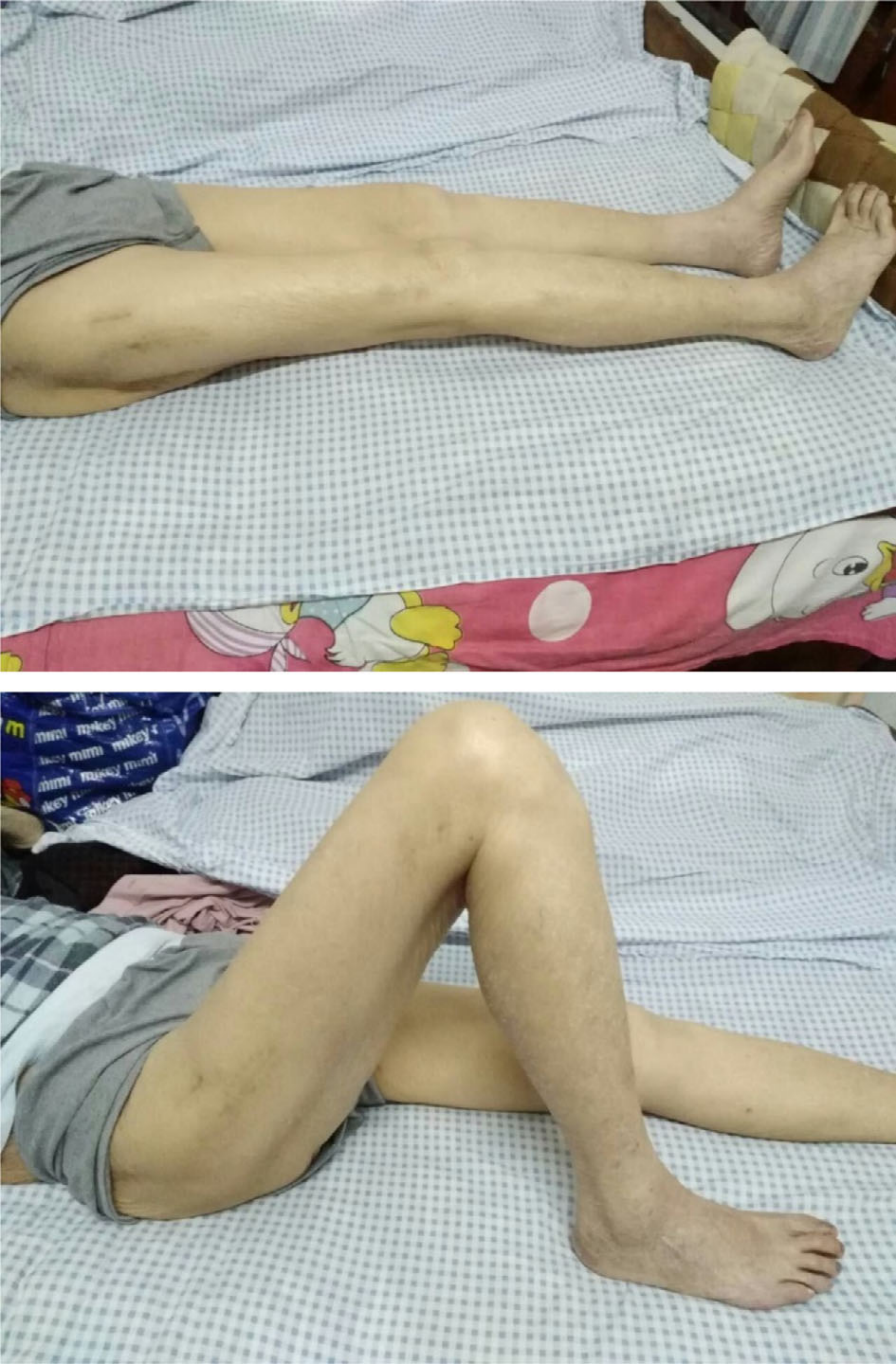
Functional results one year after surgery.

**Fig. 23:**
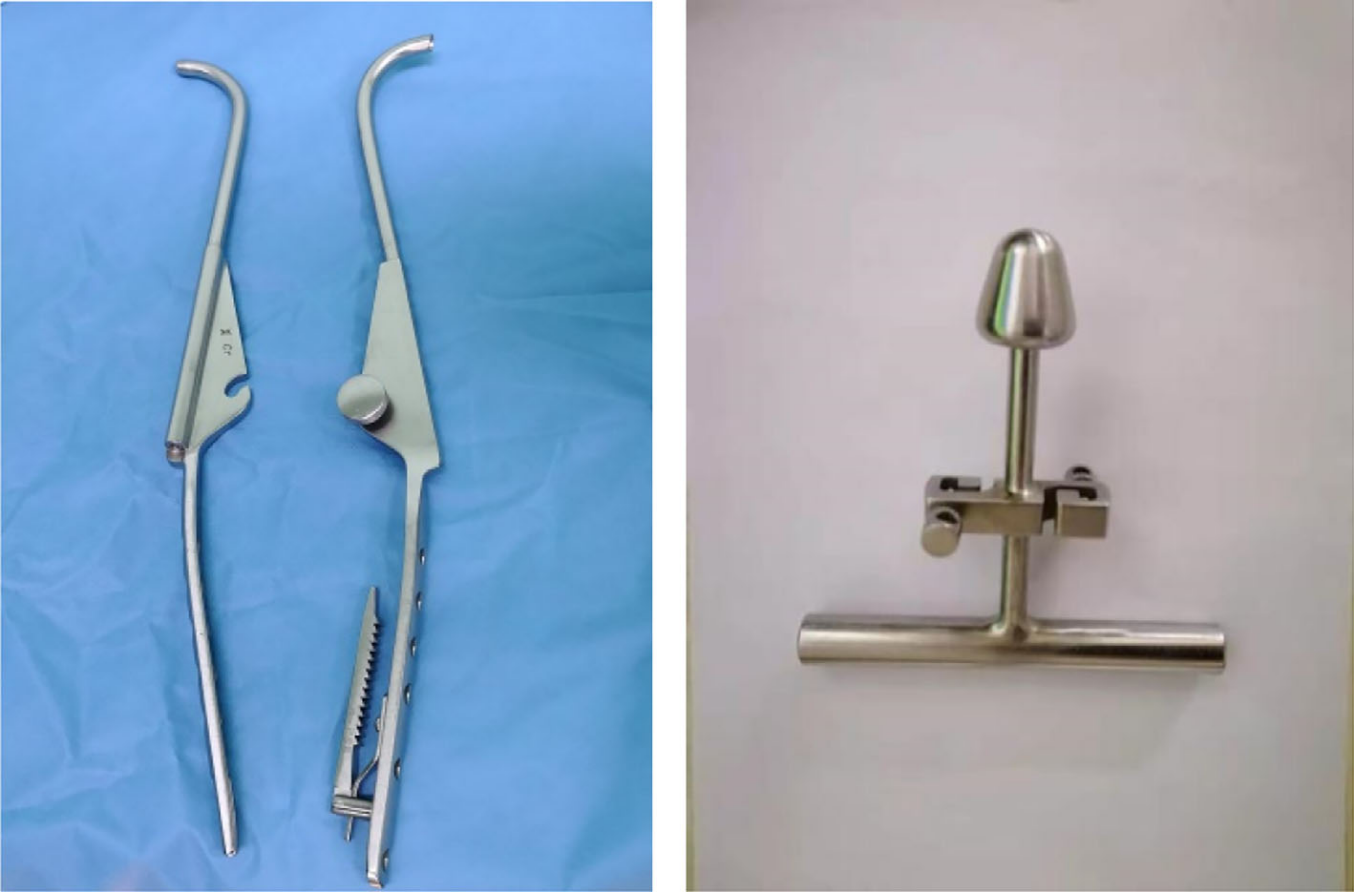
Passer and wire traction and tighting devices.

## Discussion

In this study, double‐stranded steel wires are inserted with a percutaneous wire introducer to treat irreducible SFFs, and a wire traction and tightening device is used to compress and reduce long butterfly or long spiral fracture fragment. In addition, the distance between fracture ends are reduced, and reduction is maintained. In clinical practice, the method has the following characteristics. (i) It is a minimally invasive operation that requires a small incision to insert a wire introduction and reduction device to protect the blood supply of soft tissues, which is conducive to fracture healing. (ii) The wire introduction and reduction device is inserted to maintain reduction and the normal force line and medial continuity of the proximal femur and avoid wire failure and fracture nonunion. (iii) The shrinkage of the bone fragment space can further facilitate the healing of the fracture.

### Influencing Factors for SFF Healing

Two factors affect the healing of SFFs^10^, mechanical stability and the local soft tissue blood supply. Implantation options for SFFs are divided mainly into extramedullary fixation (e.g., proximal femoral locking plates, DHS, DCS, and so on) and intramedullary fixation (e.g., femoral reconstruction nail, lengthened PFNA, and INTERTAN nail). Recently, studies have observed the advantages of intramedullary fixation over extramedullary fixation^21^ and the significantly lower failure rate of the implants of the former method. The reasons for these findings are as follows. Intramedullary fixation has the following advantages: short‐arm force, small bending moment, strong antirotation capability, superior stress dispersion, and improved overall internal fixation and bone stability.

Contralateral tensile stress increases by 60% when SFF medial cortical defect occurs and by 370% in the case of extensive defect^22^, which is the main cause of fracture instability; thus, reconstructing medial bone cortex continuity is essential. An intramedullary nail cannot fix a butterfly‐shaped or long spiral bone block owing to far‐ and near‐locking nail fixation, and a fracture end displacement of more than 1 cm or plane more than 10 degrees will lead to delayed union and nonunion^23^, ^24^. Therefore, other auxiliary internal fixators are necessary to increase inner‐side stability. Seyhan^25^ compared the effects of blocking‐screw and steel‐wire cerclage on the auxiliary reduction of SFFs. The operation time and fluoroscopy time of the blocking‐screw cerclage group are significantly longer than those of the steel‐wire cerclage group, which shows the advantages of the wire introduction‐assisted intramedullary treatment of SFFs.

Another cause of SFF nonunion is severe damage to the soft tissue blood supply. According to the traditional view, one of the reasons for failed surgeries is the complexity of fracture closed reduction. To achieve ideal reduction, open reduction is necessary. The excessive peeling of soft tissues can lead to the nonunion of fractures. For long tubular bones, wire cerclage can destroy the blood supply of the outer membrane of the bone, and multiple‐steel‐wire cerclage can lead to ischemic necrosis of the central part of the fracture, which is the main cause of complications, such as fracture nonunion, bone infection, and internal fixation failure. However, in the past 10 years, views gradually changed. Wayne *et al*.^14^ believed that anatomical reduction is key to the successful treatment of SFFs. Sanjay^26^ suggested that anatomical reduction be performed as much as possible for SFFs. Open reduction is recommended if closed reduction cannot achieve acceptable results. Krappinger^27^ considered the prevention of varus and restoration of medial continuity as the most important factors for preventing fracture nonunion.

With the increasing number of anatomical studies on the femoral blood supply and periprosthetic fractures, wire cerclage combined with implant fixation became a common treatment method^28^. The retrospective study of Codesido^29^ showed that open reduction combined with intramedullary nail fixation is better than closed reduction and intramedullary nail fixation for SFFs. The results of an anatomical study of fresh animal and human cadavers by Perren^30^ and Apivatthakakul^31^demonstrated that the blood supply of the femur is circular, and the blood supply of the femur after percutaneous wire cerclage is very small. However, this effect can be compensated by the branches of the superficial femoral artery and deep femoral artery. Joon Woo Kim^23^ used minimally invasive percutaneous wire cerclage for temporary fixation and extended proximal femur intramedullary nails to fix 12 unstable SFFs, all of which healed. The author believed that percutaneous wire cerclage has little influence on the blood supply and can achieve anatomical reduction and temporary fixation. After intramedullary nail fixation, early movement is allowed, which is conducive to fracture healing. Kilinc^17^observed 52 patients and argued that open reduction and wire cerclage have no negative effects on the healing of SFFs. Moreover, anatomical reduction and medial support can prevent internal fixation failure and nonunion. We observe that after inserting a passer in a fracture and after traction, rotation, and compression, fracture reduction can reach an ideal position, and the blood supply can be protected by narrowing the fracture space, thereby healing the fracture smoothly.

### Advantages of Minimally Invasive Wire Introduction and Screwing Traction Reduction Device

At present, commonly used auxiliary reduction implants include steel wires, steel cables, and microplates^32^. Steel cables are processed by twisting multiple strands of steel wires. Through a comparison of biomechanics, it was determined that steel cables have obvious mechanical advantages over steel wires^33^. Liu and Hu^34^ believed that in the process of fixing, steel wires can break easily from excessive pressure and slip easily if they become too loose. Therefore, the use of steel cables for fixing is recommended. In the first case in this study, we used steel cable fixation. No significant difference was observed in the surgical incision and operation time during the reduction process, insertion of the passer, and introduction of the steel cable compared with that of the steel wire. However, in the process of inserting both ends of the steel cable into the fixed lock catch, a 3 to 4 cm‐long incision could not vertically lock and fix the pressurizer; thus, extending the incision was necessary, which significantly increased soft tissue injury and peeling. The steel cable cannot achieve effective temporary reduction if the lock is not fixed firmly. To fix the wire minimally and effectively, we designed a wire screwing traction reduction pressurizer with the following advantages.

(i) The serpentine head on the front side can be pushed easily to the bone surface by a steel wire. Compared with the incision for the reduction, the surgical incision is smaller, which can reduce damage to the skin, subcutaneous tissues, muscles, and periosteum; trauma; and bleeding. Moreover, the periosteum is well protected, damage to the blood supply of the broken end does not increase, and healing is fast.

(ii) Bones are rounded, and ropes, such as steel wires, are variable. When the steel wires are tightened perpendicular to the bone surface, the steel wires will produce round‐like deformations. This deformation will generate a squeezing force and cause displacement on the internal tissues of the ring, and the displaced bones will move to the defect, that is, the fractured area. The two fracture distances can be moved closer together to achieve the reduction effect.

(iii) In the steel wire pressurization and tightening process, steel‐wire and through‐hole pressurization, locking screw tightening, and other operations can be performed independently to complete fixation to achieve the pressurizing effect on the broken end. Special tools are not needed to increase pressure and tighten the incision to achieve secondary reduction and the reduction effect.

(iv) Rotary tightening generally requires traditional tools, such as a vice, to penetrate the periosteum from the skin. In the circular soft tissue channel, the square structure of the vice will generate resistance in the placement and rotation process, which often fails to achieve the tightening effect. The serpentine head design can be quickly inserted into the periosteum without resistance during rotation, which can exert a satisfactory effect on wire tightening.

(v) Clinical observations showed that the retractor can significantly enhance the surgical effect, shorten operation time, reduce surgery risks, and obtain satisfactory surgical treatment effects. Furthermore, steel wire cerclage can promote the anatomical reduction and effective temporary fixation of fractures.

Microplate‐assisted fixation is also a commonly used method in clinical practice. The general method involves cutting the lateral thigh muscle from the anterolateral side and fixing it to a microplate. To stabilize a fracture, at least two screws are fixed to each broken end. Therefore, if the plate is placed in the anterolateral femur, then a 6 to 8 cm skin incision is necessary, and the soft tissue peeling of muscles will occur, which can increase the risk of nonunion.

From an economic perspective, compared with steel wires, steel plates and steel cables can significantly increase patients' economic burden.

Therefore, as a temporary fixation implant, double‐stranded steel wires have the following advantages: (i) can achieve the minimally invasive reduction effect and maintenance reduction; (ii) can reduce damage to soft tissues; and (iii) can reduce patient's economic burden.

### Indications for Temporary Fixation with Percutaneous Wire Introduction and Cerclage

For SFFs, especially comminuted fractures, if the injury is not serious and fracture displacement is small, then the remaining soft tissue hinges are typically used to achieve the ideal position through closed reduction. At this time, standard lengthened intramedullary nails can be used directly. For SFFs with obvious displacement, during the traction process, owing to the role of the iliopsoas and gluteal muscles, the proximal end tends to increase flexion, abduction, and external rotation displacement and thus achieving successful closed reduction is difficult. Therefore, an anterolateral incision should be made, and the fracture line should be closed with a periosteal stripper, puncture forceps, or other reduction tools. If ideal reduction can be achieved, then setting is unnecessary. A wire cerclage can be inserted, and it can be fixed with an intramedullary nail. Direct wire cerclage and fixation without compression reduction with a periosteal stripper can cause femoral vascular damage^35^, ^36^. Therefore, after clinical operation and literature research, we adopted a three‐step method for irreducible SFFs, that is, closed reduction, percutaneous tilting reduction, and percutaneous wire cerclage.

### Wire Insertion Techniques

A few tips are provided for the utilization of a wire introducer to implant and fix a wire. (i) Full traction is required before surgery. After an SFF, the contraction of quadricep muscles will lead to the shortening of the injured limb. If traction is not performed before surgery, maintaining the length of the femur during the operation and the minimally invasive reduction operation would be difficult. (ii) The displacement of the distal and proximal ends of the long wedge‐shaped or spiral‐shaped bone should be corrected during surgery. (iii) For a long wedge‐shaped or spiral fracture distance larger than 5 cm, the distal and proximal fragments of the fracture can be selected and fixed with two steel wires. During surgery, the fragments must be located accurately under fluoroscopy, and 3 to 4 cm local incisions must be made. In addition to the gap between the two incisions, a gap should be placed between the front and back to prevent the two incisions from connecting and becoming one incision, which can protect the blood supply within a maximum range. The fixation of two fractures can be reduced evenly to achieve satisfactory results; otherwise, a seesaw phenomenon will occur, in which one side is reduced satisfactorily, but the other side is tilted. (iv) The use of steel wire cerclage should be reduced if it is not necessary. De Vries^37^ reported on the use of four steel cables for fixation in a case, resulting in the nonunion of a fracture. A patient was treated *via* two‐wire fixation in our department, which resulted in the delayed union of the lateral femoral cortex.

This study has a few limitations: (i) the sample size is too small, and the follow‐up time is too short, which should be improved continuously by adopting long‐term follow‐up examinations: and (ii) this study lacked a randomized control group, which should be improved.

In summary, the combination of a minimally invasive wire introduction and wire‐traction and tighting devices with a lengthen Cephalomedullary nail is an effective clinical method for the treatment of irreducible SFFs, which has the following unique advantages. (i) The application of local wire cerclage for a fracture can restore the continuity and integrity of the medial cortex, reduce the burden of the intramedullary nail, avoid hip varus, and decrease the incidence of implant failure. (ii) The reduction of a fracture, introduction of steel wires, and final fixation of the reduction and compression are the most minimally invasive methods for clinical assisted reduction. Moreover, in such methods, operation wounds are small, and the surrounding tissues are less peeled, which can prevent blood supply damage and is conducive to fracture healing. (iii) The method can reduce patients' economic burden. Therefore, the clinical promotion of this method would be a worthy endeavor.
